# Atypical Kawasaki Disease after COVID-19 Vaccination: A New Form of Adverse Event Following Immunization

**DOI:** 10.3390/vaccines10010126

**Published:** 2022-01-16

**Authors:** Ana Lilia Peralta-Amaro, Melina Ivone Tejada-Ruiz, Karen Lilian Rivera-Alvarado, Orestes de Jesús Cobos-Quevedo, Patricia Romero-Hernández, Wiliams Macías-Arroyo, Alberto Avendaño-Ponce, Jorge Hurtado-Díaz, Olga Vera-Lastra, Abihai Lucas-Hernández

**Affiliations:** 1 Department of Internal Medicine, Hospital de Especialidades Dr. Antonio Fraga Mouret Centro Medico Nacional “La Raza”, Instituto Mexicano del Seguro Social, Mexico City 02990, Mexico; ranitaper22@gmail.com (A.L.P.-A.); melina.tejada.ruiz@gmail.com (M.I.T.-R.); orestes.cq@live.com.mx (O.d.J.C.-Q.); pat_1892@hotmail.com (P.R.-H.); willy_13126@hotmail.com (W.M.-A.); albertoap212@gmail.com (A.A.-P.); olgavera62@yahoo.com.mx (O.V.-L.); 2 Division of Post-Graduate Studies, Faculty of Medicine, Universidad Nacional Autónoma de México, Mexico City 04360, Mexico; karenlilian.rivera@gmail.com (K.L.R.-A.); jorge_hurtado01@hotmail.com (J.H.-D.); 3 Department of Rheumatology, Hospital de Especialidades Dr. Antonio Fraga Mouret Centro Medico Nacional “La Raza”, Instituto Mexicano del Seguro Social, Mexico City 02990, Mexico

**Keywords:** Kawasaki disease, medium vessels, vasculitis, AEFI, COVID-19 vaccine, SARS-CoV-2, vaccine reaction, Vaxzevria

## Abstract

Kawasaki disease (KD) is a medium-vessel vasculitis that is typically presented during childhood; fewer than 100 cases of KD have been reported worldwide in adult patients who met the criteria according to the American College of Rheumatology. This study presents the case of an 18-year-old patient with no previous history of any disease, who presented atypical KD with liver and kidney dysfunction, with a good response to intravenous immunoglobulin therapy. The symptoms began 22 days after the application of the COVID-19 vaccine (nonreplicating viral vector Vaxzevria), and other conditions were ruled out. The term Adverse Events Following Immunization (AEFI)encompasses all the reactions that follow the application of any vaccine with no necessary causal relationship and can be due to the vaccine product, quality of the vaccine, immunization errors, or anxiety or just happen to be coincident events. These reactions should be reported so that clinicians can identify compatible cases and consider that the presentation of this disease, despite being atypical, can be manifested in adult patients. Likewise, case reports are an important basis for the pharmacovigilance of vaccines.

## 1. Introduction

Since the beginning of vaccination, the incidence of new Coronavirus Disease 2019 (COVID-19) cases and mortality has radically dropped. The different vaccines that have been accepted for inoculation in the population have different mechanisms of action, based on nucleic acids (RNA and DNA), protein subunit, inactivated virus, viral vector (non-replicating), and virus-like particle, among others [1]. Several Severe Acute Respiratory Syndrome Coronavirus 2 (SARS-CoV-2) vaccines are currently used with proven safety and efficacy, among those, the non-replicating viral vector Vaxzevria [2,3,4].

Vaccines of any kind are always potentially associated with adverse events. The use of the term Adverse Events Following Immunization (AEFI) has spread to include “any adverse medical event that follows vaccination and that does not necessarily have a causal relationship with the use of the vaccine” [5,6]. The Working Group on Vaccine Safety, an organization established by the World Health Organization (WHO) and United Nations Educational, Scientific and Cultural Organization (UNESCO) in 1949 and a subgroup of the Council for International Organizations of Medical Sciences (CIOMS), proposes classifying AEFI into five groups: (1) reactions related to the vaccine product, (2) reactions related to defects in the quality of the vaccine, (3) reactions related to immunization errors, (4) reactions related to anxiety, and (5) coincident events [6]. On the other hand, the WHO published a Manual for Causality Assessment of AEFI, which includes four possibilities of immunization reactions: (A) consistent with causal association to immunization, (B) indeterminate, (C) inconsistent with causal association to immunization, and (D) unclassifiable [7].

Given the public health urgency and the extensive vaccination campaigns planned around the world, the European Medicines Agency (EMA) and the competent national authorities (NCAs) of the EU member states have prepared for the high-volume data expected by implementing this pharmacovigilance plan for COVID-19 vaccines. This is to ensure that all new information collected after marketing is immediately reviewed and that any new information that emerges is shared with the public promptly. The plan was based on the well-established pharmacovigilance system of the EU regulatory network and the experience gained during the 2009 H1N1 influenza pandemic, considering the current specificities of the COVID-19 pandemic [8].

Unfortunately, some nations do not even have this pharmacovigilance experience, which represents an area of opportunity to strengthen engagement and collaboration with stakeholders, including vaccination and healthcare professionals, to notify and recognize vulnerable populations. Thus, the principal objective for reporting this case, a Kawasaki Disease (KD) in an adult after COVID-19 vaccination, is to underline the importance to identify and describe new clinical manifestations related to COVID-19 vaccination.

## 2. Case Report

A previously healthy 18-year-old man received his first dose (31 July 2021) of the SARS-CoV2 vaccine (Vaxzevria). On the 22nd day post-vaccination, he began with fever >38.5 °C, headache, and diarrhea, followed by conjunctival injection and skin lesions on the thorax and hands. He received acetylsalicylic acid and naproxen, with partial improvement. On the 29th day post-vaccination, the patient presented sudden pain, cyanosis, and right leg coolness, and numbness, for which he was hospitalized.

**Figure 1:**
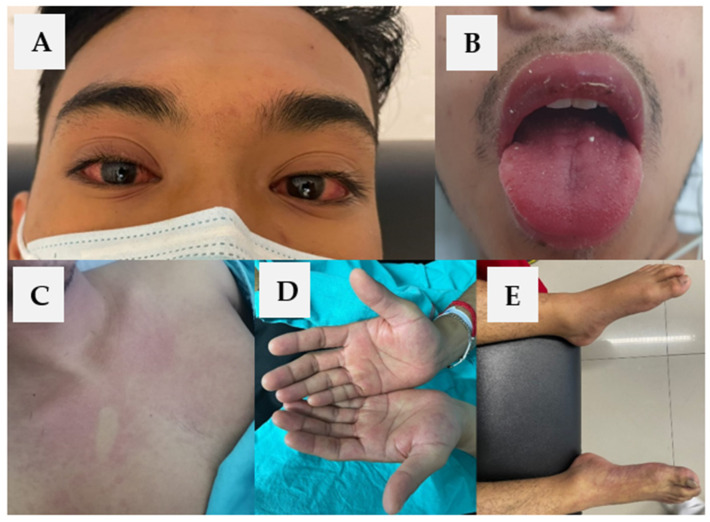
(**A**) Non-suppurative bilateral conjunctival injection, (**B**) erythema and cracking of lips, strawberry tongue, (**C**) chest morbilliform macular rash, (**D**) palmar erythema with superficial scaling, and (**E**) acute arterial insufficiency of the right foot and leg.

Physical examination confirmed a fever, non-suppurative bilateral conjunctival injection, chest and abdominal morbilliform macular rash, palmar erythema with superficial scaling, cracked and erythematous lips, strawberry tongue, jaundice, cervical lymphadenopathy, and acute arterial insufficiency of the right foot and leg (Figure 1). Infections (including COVID-19) and alternate rheumatologic diseases were ruled out. Laboratory tests showed hepatic enzymes and CRP elevation and altered markers of coagulation (Table 1). Arterial thrombosis of the right leg was diagnosed by Doppler ultrasound. Diagnosis of atypical KD with liver involvement, secondary coagulopathy, and acute kidney injury (AKI) was integrated. After 24 h of his admission, he presented a spontaneous resolution of arterial thrombosis with non-traumatic rhabdomyolysis secondary to reperfusion injury (creatine phosphokinase 20,275 IU/L). Treatment was started with a single dose of 100 g intravenous immunoglobulin (IVIg) for 12 h, solutions at 2 mL/kg/h; due to coagulopathy and thrombocytopenia, it was decided to individualize the dose of acetylsalicylic acid (ASA) to 100 mg/day. Within the first five days after starting treatment, he began to show gradual clinical and biochemical improvement. In the subacute phase, the echocardiogram and CT angiography did not show coronary aneurysms. Currently, he is under medical surveillance and rehabilitation for sensory and motor sequelae of the right limb after arterial thrombosis (Figure 1).

## 3. Discussion

We report a case of a male adult with AEFI manifested as KD that was noted after receiving COVID-19 vaccination. KD has been reported previously with the pneumococcal vaccine but not in COVID-19, until now. AEFI can be classified according to its etiology, mediated or not by the immune system. While all allergic reactions are immune-mediated, not all immune-mediated reactions are allergic. Possible causes of an allergic reaction to a COVID-19 vaccine are usually due to the reaction to adjuvants and other excipients or components in the vaccine, rather than to the active principle itself. Non-allergic reactions, which can be local, such as swelling and erythema at the injection site, can occur days after administration. On the other hand, vaccination has been associated as a possible trigger for autoimmune-autoinflammatory, or mixed disease phenotype [9].

Kawasaki disease (KD) is an acute multisystemic vasculitis, manifested by a constellation of signs and symptoms, including fever, polymorphic rash, conjunctival injection, cervical lymphadenopathy, changes in the oral mucosa such as erythema and cracking of the lips, strawberry tongue, diffuse injection of oral and pharyngeal mucosa, and characteristic changes in the extremities, such as desquamation, erythema, and edema of the palms and soles. Approximately 20% of untreated patients and 4% of treated patients develop dilatation and aneurysms of the coronary arteries. Epidemiological data suggest that the disease occurs worldwide in individuals of all races, with the highest incidence in Japan, Korea, and Taiwan and other people of Asian ethnicity [10]. KD in adults is very rare; thus the diagnostic criteria are the same as in the child population [11]. It is estimated that between 65% and 80% of cases occur between 6 months and 5 years of age, and cases are rarely reported in adults, which has led to difficulties in determining whether there are clinically relevant differences between children and adults [12]. Cheilitis, meningitis, and thrombocytosis have been reported in a higher percentage of children, while adenopathies, arthralgias, and liver function abnormalities are more common in adults [13].

The etiology of KD has not been fully clarified; the widely accepted consensus is that it arises from an abnormal and exaggerated inflammatory response to one or more environmental triggers in genetically susceptible individuals [14], a function of one or more superantigens produced by certain strains of Staphylococcus, Streptococcus, or HIV that can stimulate large numbers of T cells. These superantigens can interact directly with the major histocompatibility complex class II molecules on the surface of T cells and induce the following modifications: release of cytokines, activation of B cells and mononuclear cells, and adhesion of inflammatory cells to the endothelium, leading to vasculitis. Several infectious agents, such as Propionibacterium acnes, Rickettsia, Epstein–Barr virus, parvovirus B19, and retroviruses, have been suggested as possible etiologic agents, and they have also been observed after vaccination [15].

Population-based studies have evaluated the associations between KD and Pneumococcal Conjugate Vaccines (PCV). A non-statistically significant increased risk of KD after 13-valent PCV (PCV13) compared to PCV7 (relative risk 1.94, 95% CI 0.79 to 4.86) has been found from a Vaccine Safety Datalink study [16]. Other studies found no evidence of an association between KD and PCV13, PCV 7, or 4CMenB vaccines [17]. However, the vaccine can act as a factor or cofactor with underlying predisposing conditions related to the biological or genetic makeup of the individual and the interaction of multifactorial environmental variables [18].

SARS-CoV-2 infection is known to exacerbate known rheumatologic disease, unmask previously undiagnosed rheumatic conditions, and precipitate de novo disease, which may persist after resolution of the infection. The presence of autoantibodies has been described in COVID-19 patients; it has been estimated that approximately 30% of COVID-19 patients have at least one antibody against a nuclear antigen. Autoimmune disorders reported in the context of COVID-19 include hemolytic anemia, immune thrombocytopenia, cutaneous vasculitis, Graves’ disease, and acute demyelinating disorders of the Guillain Barré type or cranial nerve palsy. A syndrome similar to Kawasaki disease was reported for the first time in association with COVID-19 in children and called COVID-19 Associated Multisystemic Inflammatory Syndrome in children (MIS-C), characterized by clinical manifestations overlapping with myocarditis, Kawasaki disease, and staphylococcal toxic shock syndrome. Later, it was described in adults and was called Multisystemic Inflammatory Syndrome Associated with COVID-19 in adults (MIS-A) [19].

Watad et al. reported 27 cases of patients that presented an autoimmune or autoinflammatory disease after application of the COVID-19 vaccine from Israel, the United Kingdom, and the US; 17 were outbreaks of an underlying autoimmune/rheumatic disease before vaccination, and 10 of them were new-onset immune-mediated diseases [20]. Vera-Lastra et al. reported two cases of Graves’ Disease after COVID-19 vaccination in previously healthy adults [21].

MIS has been described as an entity that presents gastrointestinal, cardiovascular, and skin symptoms, in addition to an acute or past SARS-CoV-2 infection and is recently related after vaccination. Recently, Sharma et al. proposed that the mechanism of SARS-CoV-2 S protein acts as a superantigen that triggers the cytokine storm [22]. The difference between KD and MIS can be difficult to determine because there is not a biomarker to any of these; there are epidemiological, clinical, and immunological differences, but in medical practice, this can represent a diagnostic challenge. This patient met the criteria for KD according to the American College of Rheumatology, which requires fever for at least five days, plus four of the following, in the absence of an alternative diagnosis: (1) non-suppurative bilateral conjunctival injection; (2) polymorphous exanthema; (3) erythema and cracking of lips, strawberry tongue, diffuse injection of oral, and pharyngeal mucosae; (4) scaling, erythema, and edema of palms and soles and (5) cervical lymphadenopathy (>1.5 cm). When a patient meets the criteria and presents other clinical manifestations such as renal impairment (AKI, proteinuria, nephritis) or hepatic impairment (hepatitis with or without jaundice and acalculous cholecystitis), it is known as atypical KD [23].

There have been fewer than 100 reported cases of KD in adults worldwide [11], and the presence of atypical manifestations such as in this patient is extraordinary. The main point to treat KD in the first days of presentation is to minimize the risk of coronary artery aneurysm formation, which peaks two to four weeks after illness onset. Patients should receive high-dose IVIG at 2 g/kg over 10–12 h as well as high-dose ASA (80 mg/kg/day to 100 mg/kg/day divided every six hours) until the patient has been afebrile for over 48 h. ASA should be continued, but the dosing can be decreased to 3–5 mg/kg and maintained until there is no longer any evidence of cardiac changes about 6 to 8 weeks after illness onset [23]. As we mentioned before, we individualized the ASA dose, since there was coagulopathy and elevation of liver enzymes.

Ueda et al. reported the case of a 37-year-old female patient who met the criteria of KD, who was found to have concurrent Coxsackievirus A4 infection. She was treated like any other KD; nevertheless, since the patient presented liver dysfunction, antiplatelets were reduced and eventually suspended and changed to cilostazol due to an increase in alanine aminotransferase, as in our case, in which we it decided that the ASA dose be lowered due to the benefit versus risk from coagulopathy. After treatment with IVIg, renal function became normal (Cr: 0.9), and liver function, as well as leukocytosis, was gradually improved during hospitalization [24]. This patient had a good response after the first dose, and no cardiac alterations were found in echocardiography.

The relevance of this case is that KD in adults is rare, the renal and hepatic involvement rule-in an atypical KD, and the onset of the autoimmune-autoinflammatory phenotype AEFI was noted after COVID-19 vaccination. We consider this case as KD because of the absence of previous or concomitant SARS-CoV-2 infection and the time between the vaccination and the beginning of the symptom is undeniable. We do not consider MIS-A as the diagnosis, because of the lack of gastrointestinal (diarrhea, vomiting, abdominal pain, ascites, ileitis, colitis) and cardiovascular (myocarditis, shock) involvement.

This AEFI was classified as indeterminate with causal association to immunization, since there is a temporal relationship, and there is no definitive evidence for the vaccine causing the event. Since the secondary reaction presented by the patient was serious, he did not accept the second dose of the vaccine. In this case, the evaluation of causality is classified as possible, as it has a reasonable temporality relationship; however, as we mentioned, the etiology of KD is not completely known, so there could be different triggering factors [7]. As far as we know, this is the first case of atypical KD in an adult that complies with a temporal relationship after Vaxzevria vaccination.

## 4. Conclusions

Despite efforts to combat the COVID-19 pandemic, vaccination campaigns around the world have not ended. During the development phase of COVID-19 vaccines, a limited number of selected participants were included in clinical trials and followed up for a relatively short duration under controlled conditions. As a result, certain side effects, particularly rare or very rare ones, only emerged during real-life use in many different people. The prompt detection and evaluation of new information on the benefit–risk balance of these vaccines, timely communication, and a high level of transparency, will be key to protecting public health and ensuring the public’s trust in the vaccines and the regulatory system. It is therefore essential to closely monitor the safety and effectiveness of any medicine after it is authorized.

On the other hand, the diagnosis of KD is extremely rare in adults, and atypical symptoms are observed in few cases. Systemic manifestations after the application of vaccines are rare; nevertheless, they must be reported for further investigation. To the best of our knowledge, this is the first case described of atypical KD after COVID-19 vaccination.

AEFI dissemination should continue to be implemented in a broader way, which will allow expanding the knowledge of the diversity of clinical manifestations with the different COVID-19 vaccines. These reactions, which occur in a small percentage of the vaccinated population, should not be a contraindication or a reason to avoid vaccination. In the report made by Stefanizzi about the post-marketing surveillance of the meningococcal B vaccination, they found that from the 807,446 doses applied, 214 AEFIs were reported and only a very small group had serious reactions, so the benefit of the application of the vaccine was much higher against the risk of meningococcal meningitis [25]. We are currently in the middle of a pandemic with millions of deaths, and the application of the vaccine should be a priority for the population given the risk versus the benefit.

We encourage clinicians to recognize potential outbreaks that are in line with our reported experience. The results reassure the public that such events are rare and manageable and reassure clinicians that conventional therapies such as glucocorticoids are often appropriate.

## Figures and Tables

**Table 1:** Blood Tests.

Blood Test	Value	Reference Ranges
Kidney Function		
Creatinine	2.7 mg/dL	0.5–0.9 mg/dL
Liver Function		
Lactate dehydrogenase (LDH)	1069 U/L	300 U/L
Alanine aminotransferase (ALT)	155 U/L	13–40 U/L
Aspartate aminotransferase (AST)	286 U/L	15–48 U/L
GGT	688 U/L	250 U/L
Total Bilirrubin	14.9 mg/dL	0.2–1.2 mg/dL
Direct Bilirrubin	11.7 mg/dL	0.0–0.3 mg/dL
Albumin	2.6 g/dL	3.5–4.5 mg/dL
Coagulation Tests		
Prothrombin time (PT)	28.8 s	11 s
Partial thromboplastin time (PTT)	35.6 s	33 s
Complete Blood Count		
Hemoglobin	14.9 g/dL	12–18 g/dL
Platelets	39,400 per mL	150,000–450,000 per mL
Leukocyte	19.6 K/mcl	4.5–10 K/mcl
Neutrophils	88.2%	50–70%
Lymphocytes	2.8%	17–45%
Monocytes	2.5%	4–12%
Eosinophils	5.9%	1–4%
Basophils	0.5%	1–2%
C-reactive protein (CRP)	80.8 mg/dL	0.00–5.00 mg/dL
Autoimmune Tests		
Antinuclear antibodies (ANA)	1:80	Negative
Anticardiolipin IgM, IgG antibodies	Negative	Negative
Lupus Anticoagulant (LA)	Negative	Negative
Serological Markers		
HIV	Negative	Negative
Hepatitis B (HBV)	Negative	Negative
Hepatitis C (HCV)	Negative	Negative

Laboratories: showing elevation of liver enzymes and altered markers of coagulation. After receiving treatment with IVIg renal function became normal (Cr: 0.9) and liver function was gradually improved during hospitalization as well as leukocytosis.
